# Osteoblastic Differentiation of Human Adipose-Derived Mesenchymal Stem Cells on P3HT Thin Polymer Film

**DOI:** 10.3390/jfb16010010

**Published:** 2025-01-02

**Authors:** Paola Campione, Maria Giovanna Rizzo, Luana Vittoria Bauso, Ileana Ielo, Grazia Maria Lucia Messina, Giovanna Calabrese

**Affiliations:** 1 Department of Chemical Sciences, University of Catania and CSGI, Viale A. Doria 6, 95125 Catania, Italy; paola.campione@phd.unict.it; 2 Department of Chemical, Biological, Pharmaceutical and Environmental Sciences (ChiBioFarAm), University of Messina, Viale F. Stagno d’Alcontres 31, 98166 Messina, Italy; mariagiovanna.rizzo@unime.it (M.G.R.); luanavittoria.bauso@unime.it (L.V.B.); ileana.ielo1@unime.it (I.I.)

**Keywords:** bone tissue engineering, semiconductive polymers, cytocompatibility, osteoconductivity, osteoinductivity

## Abstract

Bone defects restoration has always been an arduous challenge in the orthopedic field due to the limitations of conventional grafts. Bone tissue engineering offers an alternative approach by using biomimetic materials, stem cells, and growth factors that are able to improve the regeneration of bone tissue. Different biomaterials have attracted great interest in BTE applications, including the poly(3-hexylthiofene) (P3HT) conductive polymer, whose primary advantage is its capability to provide a native extracellular matrix-like environment. Based on this evidence, in this study, we evaluated the biological response of human adipose-derived mesenchymal stem cells cultured on P3HT thin polymer film for 14 days. Our results suggest that P3HT represents a good substrate to induce osteogenic differentiation of osteoprogenitor cells, even in the absence of specific inductive growth factors, thus representing a promising strategy for bone regenerative medicine. Therefore, the system provided may offer an innovative platform for next-generation biocompatible materials for regenerative medicine.

## 1. Introduction

Traumatic injuries to bone tissues can be caused by a wide range of events, including falls, accidents, violent impacts, and recurrent stress. These have long-term effects that go beyond temporary pain and impairment, and they frequently result in disability, a reduced quality of life, and functional restrictions [1,2]. Moreover, bone injuries, such as fractures and traumatic damage, can exacerbate underlying conditions, like osteoporosis and osteoarthritis. Addressing the challenges posed by bone diseases requires innovative approaches that go beyond conventional treatments.

Recently, the tissue engineering has provided a new approach for bone repair that is able to address all clinical requirements; overcome drawbacks including donor site morbidity, pain, rejection, transmission of diseases, high cost; and improve healing [3,4]. The bone tissue engineering (BTE) strategy makes use of biomaterials, progenitor cells, and growth factors, with the aim of developing scaffolds capable of mimicking natural bone tissue and improving osteoregenerative properties by enabling or improving host integration, adhesion, cell viability, growth, differentiation, and vascularization, thus reducing the limitations of conventional grafts [5,6].

Biomaterials play a crucial role in the multistage process of cell adhesion, which includes the attachment, spreading, and formation of stress fibers and focal adhesions at very early stages [7]. As is well known, biomaterials’ bulk properties are critical, but their surface properties are even more important, because they aid in the design of functional materials and the promotion of cellular processes including protein adsorption, recolonization, recruitment, attachment, proliferation, migration, and immune-inflammatory responses [8]. Thus, regulating the biomaterial surface properties is a crucial challenge in planning innovative smart substrates and regenerative approaches [9].

Surface properties, including topography, stiffness, surface free energy (SFE), roughness, and chemistry, can drive cell fate since it is regulated by both biochemical and biophysical signals, i.e., it is possible to modify actin remodeling, focal adhesion, and integrin clustering, which in turn alter mechanosensitive signaling cascades involved in the development of cells [10,11,12,13,14,15].

A variety of biomaterials, including metals, composites, hydrogels, natural and synthetic materials, ceramics and polymers, have been employed in the creation of scaffolds [16,17]. In recent years, electroactive conductive polymers have also attracted more attention for tissue engineering and bone repair [18,19,20,21].

Among these, poly(3-hexylthiofene) (P3HT), a semiconducting polymer with intriguing electronic and structural properties, has garnered attention in recent years. Beyond its traditional applications in optoelectronics devices and solar cells, P3HT holds considerable promise in the field of tissue engineering, thanks to its unique mechanical and electrical properties. It serves as a dynamic platform that can enhance responsive tissue scaffolds, facilitating essential cellular processes such as adhesion, proliferation and differentiation [22,23,24]. One of the primary advantages of P3HT is its ability to provide a growth environment like the native extracellular matrix [25,26,27]. Moreover, the conductive properties of the material make it especially attractive for modulating cell responses via electrical signaling, in fact it can be used to induce the neurogenesis through electrical or optoelectrical stimulation [28,29,30]. The intrinsic conductivity characterizing P3HT actively participates in cell signaling. Recent studies have demonstrated that P3HT integration into scaffolds can significantly affect osteogenic differentiation by mimicking the electrical signals that occur in natural bone tissue, leading to enhanced bone and muscle tissue formation [25,31]. When mesenchymal stem cells are cultured on conductive materials, such as P3HT, electrical cues can promote the activation of specific transcription factors associated with osteogenic differentiation pathway and can affect gene expression. The conductive properties of materials can enhance ion transport and create electrochemical gradients that affect the cellular microenvironment [32,33]. This interplay fosters communication between electrical signals and biological tissues, promoting the expression of osteoblast-specific markers and facilitating the process of matrix mineralization. Bone tissue is recognized for its piezoelectric properties, which enable the conversion of mechanical forces into electrical potential. This means that both mechanical stress and electrical signals directly affect bone formation and remodeling [34,35]. In this regard, P3HT creates a more biomimetic environment due to its mechanical characteristics, boasting an elastic modulus that closely matches that of bone tissue, as well as its native conductivity, which is like that of the biological material, enhancing its use in regenerative medicine and bone tissue engineering [36].

In this study, we investigate the properties of P3HT thin polymer film to promote the osteogenesis of human adipose-derived mesenchymal stem cells (hADMSCs). P3HT, acting as a bioactive stimulus, can modulate gene expression in stem cells, facilitating their differentiation into the osteogenic phenotype.

## 2. Materials and Methods

### 2.1. Materials and Substrates

All solvents and chemicals were of reagent quality and used without further purification. Chloroform and ethanol were obtained from Merck (Darmstadt, Germany).

Silicon (100) wafers (Siegert) and cover glasses were used as substrates. The glass surfaces were cleaned individually by sonication in ethanol for 30 min, then treated for 30 min in a UV/O_3_ chamber (UV/Ozone ProCleaner, BioForce Nanosciences, Wetzlar, Germany), rinsed with Milli-Q water, and dried with nitrogen. Silicon substrates were subjected to 15 min of UV/O_3_ treatment, rinsed with Milli-Q water, and gently dried with nitrogen.

Regioregular poly-3-hexylthiophene (P3HT) with an average molecular weight of 50,000–100,000 was purchased from Merck (Darmstadt, Germany). Thin layers of polymer P3HT film were obtained by spin-coating solutions with a concentration of 2.5 mg/mL in chloroform at 3500 rpm for 60 s.

### 2.2. Physico-Chemical Characterization by Atomic Force Microscopy

The experimental apparatus used for these measurements was NTEGRA AFM (NT-MDT, Moscow, Russia) equipped with stiff, single-crystal silicon cantilevers with a symmetric tip shape (model Tap300Al-G, BudgetSensors, Sofia, Bulgaria) showing the following specifications: nominal frequency of 300 kHz, nominal spring constant 40 Nm^−1^, and a tip radius < 10 nm. Before every measurement, the probe was characterized by quantifying the cantilever spring constant by the Sader method [37], and calibrated by performing a force curve on a <100> silicon wafer hard substrate to calculate the sensitivity. The Young’s modulus was then calculated from the collected force-distance curves by applying the Derjaguin–Müller–Toporov (DMT) model [38], using the following equation:

$$F+F_{\mathrm{ad}}=\frac{4E_{s}}{3\left(1-v_{s}^{2}\right)}R^{\frac{1}{2}}\delta^{\frac{3}{2}}$$

where F is the applied force; F_ad_ is the adhesion force; E_s_ is the Young’s modulus; v_s_ is the sample’s Poisson’s ratio; R is the radius of the spherical indenter; and δ is the depth of the elastic indentation. Different areas of each surface of the samples were indented and about three hundred curves were captured. The measures were achieved in triplicate. To determine Young’s modulus the experimental curves were fitted to the DMT model in the elastic area. The obtained results were examined using Origin 2018 software (OriginLab Corporation, Northampton, MA, USA) to assess the elastic modulus distribution.

Morphological analysis at the nanometer scale was performed in tapping mode using a Nanoscope IV-MultiMode AFM from Digital Instruments (located in Santa Barbara, CA, USA).

The instrument featured a scanner <J> that was calibrated with the help of grating manufacturers. Images were captured at a scan rate of 1 Hz, achieving a high resolution of 512 × 512 pixels per image. This was accomplished using n-doped silicon cantilevers from BudgetSensors, which had a nominal force constant of 40 N/m and a resonant frequency of 300 kHz. The amplitude setpoint was calibrated to about 75% of the free oscillation amplitude to reduce interaction between the tip and the sample. Image analysis was performed using DI software, version 5. 30, during which the images were flattened to eliminate any background slopes.

### 2.3. Biological Analyses

#### 2.3.1. Experimental Groups

To evaluate the osteo-conductive/-inductive properties of the polymeric film, we performed all the biological analyses on hADMSCs cultured in two different culture conditions (normal growth medium (GM) and osteogenic differentiation medium (OM)), and on two different surfaces (P3HT coated and uncoated slides).

Specifically:

1. Uncoated slide in GM (CTRL GM);
2. Uncoated slide in OM (CTRL OM);
3. P3HT-coated slide in GM (P3HT GM);
4. P3HT-coated slide in OM (P3HT OM).

CTRL GM and CTRL OM are used as reference groups to evaluate whether P3HT coating per se is able to induce osteogenic differentiation (P3HT GM) and if the presence of osteo-inductive factors further enhances the process when in combination with the polymeric film (P3HT OM).

#### 2.3.2. hADMSCs Cell Culture

The human adipose-derived mesenchymal stem cells (hADMSCs) used in this study were obtained from fresh adipose tissue, as previously described in [39]. Briefly, adipose tissue, supplied by the Mediterranean Institute of Oncology (Viagrande, Italy; IOM Institutional Review Board protocol project ID code: 829_1 of 8 February 2013), was mechanically dissociated into smaller fragments and enzymatically processed with a collagenase I solution (Gibco, Thermo Fisher Scientific, Waltham, MA, USA) at 37 °C for two hours. The digested tissue was then centrifuged, the floating fat was eliminated, and the suspension was filtered and centrifuged again. Finally, the cell pellet was resuspended in a basal medium (MesenPRO RS^TM^ medium, Thermo Fisher Scientific, NYSE:TMO) containing 2.5 mM L-glutamine (Merck Life Science S.r.l., Milan, Italy), 10% FBS (Merck Life Science S.r.l., Milan, Italy), and 1% PSA (Merck Life Science S.r.l., Milan, Italy), and maintained in a humidified 5% CO_2_ incubator at 37 °C. The culture medium was replaced every 2–3 days, until the hADMSCs reached approximately 80–90% confluence.

#### 2.3.3. Cell Viability of hADMSCs Cultured on P3HT Thin Polymer Film

The cytocompatibility of P3HT thin polymer films was evaluated using a colorimetric MTT (3-(4,5-dimethylthiazol-2-yl)-2,5-diphenyltetrazolium bromide) assay performed on hADMSCs cultured on them, both in the presence and absence of osteoblastic differentiation factors. Specifically, 5 × 10^4^ hADMSCs suspended in 100 μL of MesenPRO RS^TM^ medium were slowly seeded onto slides coated with P3HT thin polymer films (P3HT sample) and uncoated slides (CTRL) and incubated in 24-well culture plates at 37 °C for 2 h. Subsequently, 1.9 mL of MesenPRO RS^TM^ medium was added, and the cells were incubated at 37 °C in a humidified atmosphere containing 5% CO_2_. After 24 h (day 0), the MesenPRO RS^TM^ medium was replaced with either osteogenic differentiation medium (OM) (StemPRO^TM^ osteogenesis differentiation kit, Thermo Fisher Scientific, NYSE:TMO) or normal growth medium (GM). The OM/GM was completely replaced twice a week for 14 days. At the end of each timepoint, the OM/GM was removed from each well, replaced with 1 mg/mL of MTT solution, and incubated at 37 °C in presence of 5% CO_2_ for 2 h. The MTT solution was then removed, and the formazan crystals were resuspended in DMSO. After incubation, the plates were read at 540 nm, using a synergy HT plate reader (BioTek Instruments, Inc., Tigan Street Winooski, VT, USA). All biological tests were performed in triplicate, and changes in cell viability were evaluated using a one-way ANOVA with the Holm test as a post hoc multiple comparison. The percentage of cell viability was calculated according to the following equation:

$$\mathrm{cell}\;\mathrm{viability}\;\left(\%\right)=\frac{\mathrm{Abs}\text{\_}\mathrm{sample}}{\mathrm{Abs}\text{\_}\mathrm{control}}\times 100$$

where Abs_sample is the absorbance of each tested sample P3HT GM, CTRL_OM, and P3HT OM; OD_control is the absorbance of the CTRL GM. MTT values were presented as mean ± SD percentage values.

#### 2.3.4. Osteoblastic Differentiation of hADMSCs on P3HT Thin Polymer Film

For osteoblast differentiation, 5 × 10^4^ hADMSCs suspended in 100 μL of MesenPRO RS^TM^ medium were gradually seeded onto the P3HT thin polymer films and incubated for 2 h at 37 °C. Subsequently, 1.9 mL of MesenPRO RS^TM^ medium was added, and cells were incubated at 37 °C in a humidified atmosphere containing 5% CO_2_.

After 24 h (day 0), the MesenPRO RS^TM^ medium was replaced with OM/GM to induce osteoblast differentiation. The media were completely replaced twice a week until 14 days.

#### 2.3.5. Extracellular Matrix Mineralization on P3HT Thin Polymer Film

The evaluation of the extracellular matrix mineralization of hADMSCs on P3HT thin polymer films was assessed by Alizarin Red S (AR S) staining. For AR S staining, the solution was prepared according to the manufacturer’s protocol and carried out as reported in [40,41]. Briefly, hADMSCs cultured on P3HT thin polymer films were fixed in 4% PFA for 15 min at room temperature, washed with H_2_O, and incubated with a 2% AR S solution for 5 min. Afterward, the slides were washed several times to remove excess staining solution, and finally mounted. The stained slides were analyzed using a Leica DMI 4000B microscope (Leica Microsystems S.r.l., Milan, Italy), and the integrated density (IntDen) was quantified using Fiji image J2 software version 2.9.0/1.54f. At least three slides per sample were analyzed.

#### 2.3.6. Gene Expression Profile of hADMSCs on P3HT Thin Polymer Film

To evaluate the mRNA levels of specific osteoblast genes, such as Osteopontin (also known as secreted phosphoprotein 1, Spp1), Osteocalcin (also known as bone gamma-carboxyglutamic acid protein, Bglap), and Osteonectin (also known as secreted protein acidic and rich in cysteine, SPARC), we performed a quantitative real-time PCR (qRT-PCR) on cells cultured on P3HT thin polymer films for 1, 7 and 14 days. Cellular RNA was isolated using the Trizol Reagent (15596026, Life Technologies, Carlsbad, CA, USA), in accordance with the manufacturer’s instructions. The Nanodrop 1000 Spectrophotometer (Thermo Fisher, Waltham, MA, USA) was used to determine the quantity and quality of RNA. An amount of 1 µg of RNA from each sample was reverse-transcribed using ImProm-II Reverse Transcription System (A3800, Promega, Milan, Italy) to obtain cDNA. Quantitative real-time PCR (qRT-PCR) was performed using a 7900 Fast Real-Time PCR System with Sso Advanced Universal SYBR1 Green Supermix (2X) (1725271, Bio-Rad Laboratories, Hercules, CA, USA). Specific osteogenic genes were evaluated, and primer sequences were designed on exon–exon junctions of the target region using primer blast, as already reported in [42,43]. Each sample was analyzed in triplicate, and the expression quantification was performed by the 2^−ΔΔCt^ method. Data were shown as fold change compared to the CTRL GM and normalized to the levels of Glyceraldehyde 3-Phosphate Dehydrogenase (GAPDH). Primer sequences are described in Table 1.

#### 2.3.7. Immunofluorescence Analysis of hADMSCs on P3HT Thin Polymer Film

To further evaluate the osteogenic differentiation of hADMSCs on P3HT polymer films, we performed an immunofluorescence analysis. First, hADMSCs on PH3T were fixed in 4% paraformaldehyde (PFA), rinsed briefly in phosphate-buffered saline (PBS), permeabilized in 0.5% Triton X-100/PBS, and blocked with 5% bovine serum albumin (BSA)/0.3% Triton X-100/PBS. Then, cells were incubated overnight at 4 °C with a rabbit anti-human antibody against Osterix, also known as Sp7 (1:500; Abcam, Cambridge, UK). After washing, cells were incubated with the anti-rabbit Alexa Fluor-488 antibody (Abcam, Cambridge, UK) at a dilution of 1:1000 for 1 h at room temperature. Then, P3HT films, containing hADMSCs, were mounted on a mounting medium with DAPI (4′,6-diamidino-2-phenylindole) (Abcam, Cambridge, UK) to counterstain the nuclei. Digital images were acquired at a 20× magnification, using a NeXcope NIB 620FL inverted microscope (TiesseLab, Milan, Italy).

#### 2.3.8. Statistical Data Analysis

Data analyses were performed as either raw data or as mean ± standard error (SE), as appropriate. One-way or multi-way ANOVA, and a post hoc Holm test was used to evaluate significant differences between several timepoints of hADMSCs cultured on P3HT thin polymer films. A *p* < 0.05 was considered statistically significant.

## 3. Results and Discussion

### 3.1. Atomic Force Microscopy Analysis of P3HT Thin Polymer Film

**Figure 1:**
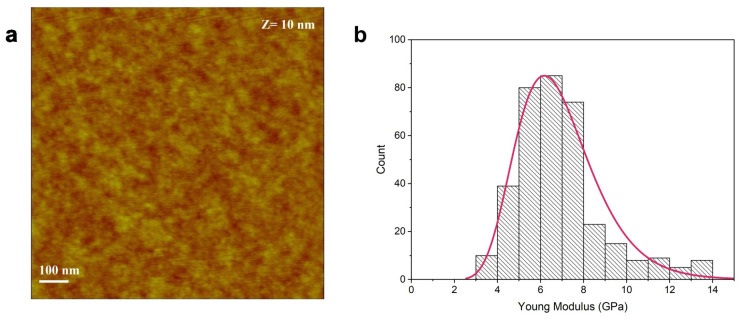
Morphological analysis (**a**) and Young’s modulus (**b**) of P3HT thin film.

Figure 1a reports the nanoscale morphology of the P3HT thin polymer film deposited on a silicon substrate. The height image highlights a homogeneous surface coverage, and the roughness appears to be substantially uniform, with a value of 0.38 ± 0.09 nm. Figure 1b shows the elastic response of the film, measured from the force–distance curves by fitting the slope in the elastic region with the DMT model, and is reported as a distribution of Young’s modulus values. The obtained values follow a LogNormal distribution, with the highest value at approximately 6.9 GPa.

### 3.2. Biocompatibility Evaluation of hADMSCs Cultured on P3HT Thin Polymer Film

**Figure 2:**
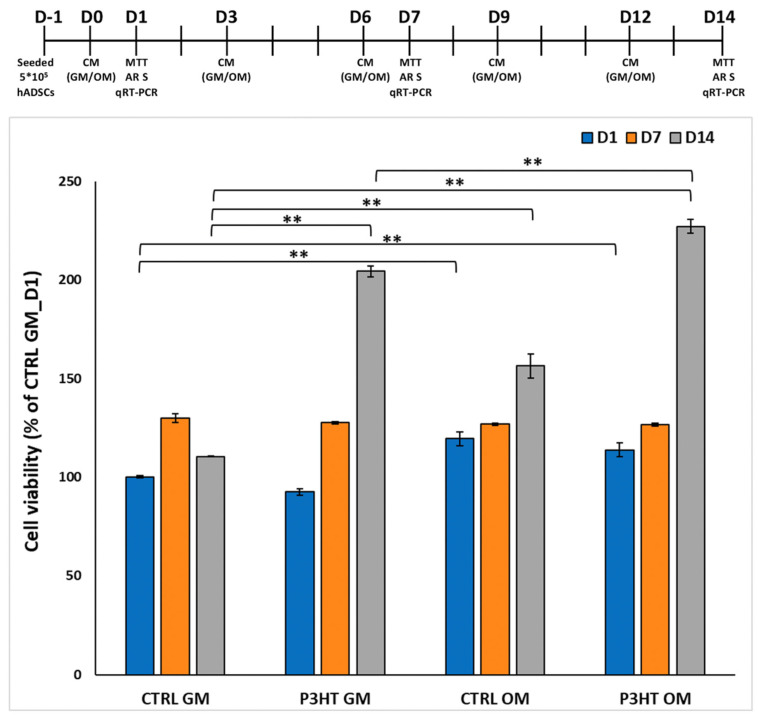
Cell viability analysis of hADMSCs cultured on P3HT thin polymer films, in GM and OM, for 1, 7, and 14 days. Data are reported as percentage of mean ± standard deviation obtained from three different samples for type. ** *p* < 0.01 show significant differences between the different groups, as reported by the Holm post hoc test.

To evaluate the biocompatibility of P3HT thin polymer films, we performed a cell viability test on hADMSCs cultured on them for 1, 7, and 14 days. MTT data, reported in Figure 2, show that the number of viable cells increase over time, from day 1 (D1) to day 14 (D14), in all analyzed samples and in both culture conditions (GM and OM) used, although the cells grown on P3HT thin polymer films exhibited higher proliferation, especially in the presence of OM. Specifically, the percentage of viable cells in P3HT-coated slides, in both culture conditions, increased 2-fold at D14, compared to D1.

These data indicate that P3HT thin polymer films are cytocompatible and promote cell adhesion and proliferation in both culture conditions used, which is consistent with other evidence in the literature [22,44]. It has been demonstrated that conductive polymers (e.g., polypyrrole (PPy), polyaniline (PANI), polythiophene (PTh), poly(3,4-ethylenedioxythiophene) (PEDOT) and polyethyleneimine (PEI)) exhibit good biocompatibility and the ability to support the in vitro adhesion, growth, and differentiation of a wide range of cell types, including osteoblasts [45], fibroblasts [46], neuroblastoma cells [47], neural cells [48], endothelial cells [49], keratinocytes [50], and mesenchymal stem cells [51].

### 3.3. Osteoblastic Differentiation of hADMSCs Cultured on P3HT Thin Polymer Film

**Figure 3:**
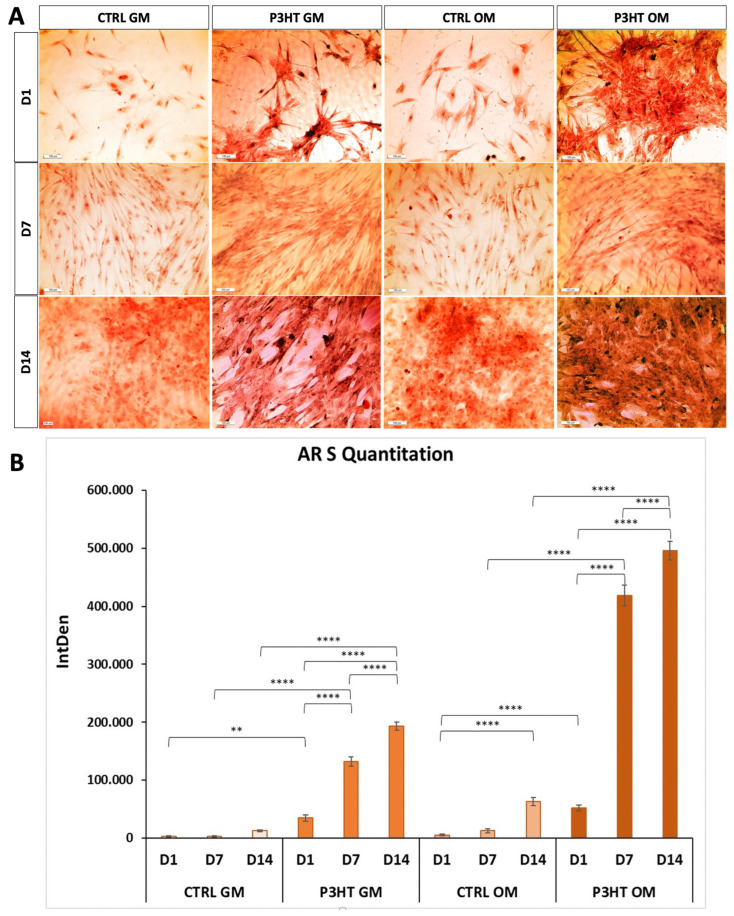
(**A**) Representative images of Alizarin Red S staining on hADMSCs cultured on P3HT thin polymer film in presence of GM or OM, for 1, 7, and 14 days. Scale bars: 100 μm. (**B**) Quantitative evaluation of the Alizarin Red S images by measuring the integrated density (IntDen). The multi-way ANOVA test *p*-value is reported, and ** *p* < 0.01 and **** *p* < 0.0001 show significant differences between the different groups, as reported by the Holm post hoc test.

To evaluate the extracellular matrix mineralisation of hADMSCs cultured on P3HT thin polymer films, we analyzed the presence of calcium deposits by Alizarin red S (AR S) staining. AR S staining was performed after 1, 7, and 14 days of cell growth in the presence of GM or OM. Data reported in Figure 3 display an increase in mineralisation over time, from day 1 to day 14, for both P3HT GM and P3HT OM conditions, compared to the controls, even though the presence of the differentiation medium induced a higher production of calcium deposits in P3HT OM compared to P3HT GM (Figure 3A,B). More specifically, AR S quantification displayed an increase in calcium deposition of approximately 3.8-fold (D7 vs. D1) and 5.6-fold (D14 vs. D1) for P3HT GM, 8.1-fold (D7 vs. D1) and 9.6-fold (D14 vs. D1) for P3HT OM, and approximately 1.5-fold at D1, 3.2-fold at D7, and 2.6-fold at D14 for P3HT OM compared to P3HT GM (Figure 3B). The above data indicate that P3HT polymeric thin films can induce the matrix mineralization of hADMSCs, even without a specific osteogenic differentiation medium, although its presence increases calcium deposition.

**Figure 4:**
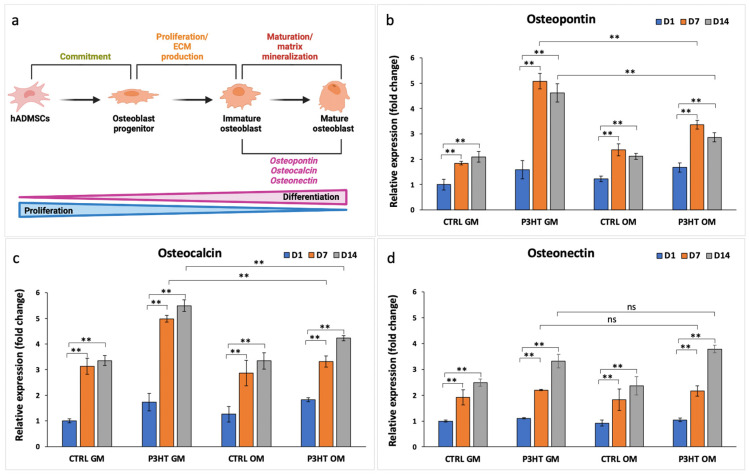
(**a**) Representative diagram of osteogenic markers expression during the osteoblastic differentiation of hADMSCs. Gene expression profile of (**b**) Osteopontin, (**c**) Osteocalcin, and (**d**) Osteonectin in hADMSCs cultured on uncoated (CTRL) and P3HT-coated slides, in presence of GM or OM for 1, 7, and 14 days. Glyceraldehyde-3-phosphate dehydrogenase (GAPDH) was used as an endogenous control. The multi-way ANOVA test *p*-value is reported, and ** *p* < 0.01 show significant differences between the different groups, as reported by the Holm post hoc test. ns = not significant.

To further evaluate the osteoblastic differentiation capability of hADMSCs cultured on P3HT thin polymer films, in the presence/absence of osteogenic differentiation medium (OM/GM), we performed qRT-PCR and immunofluorescence analysis with specific osteoblastic differentiation markers: Osterix and Osteopontin, involved in the initiation step, and Osteocalcin and Osteonectin, involved in the maturation step of matrix mineralization [52]. The qRT-PCR data shown in Figure 4 display that the expression levels of all the analyzed markers of osteoblastic differentiation increased over time, from day 1 to day 14, in all groups examined, although hADMSCs cultured on P3HT thin polymer films showed increased target modulation.

Furthermore, Osteopontin and Osteocalcin expression levels in hADMSCs grown on P3HT GM were higher compared to the cells grown on P3HT OM. Specifically, the Osteopontin levels of hADMSCs cultured on P3HT GM were approximately 1.5–1.6-fold higher at D7 and D14, respectively, than those of the cells grown on P3HT OM, while the Osteocalcin levels increased by approximately 1.5–1.3-fold. However, Osteonectin levels were similar for both cells grown in P3HT GM and P3HT OM and in those grown on CTRL slides in both culture media (Figure 4).

**Figure 5:**
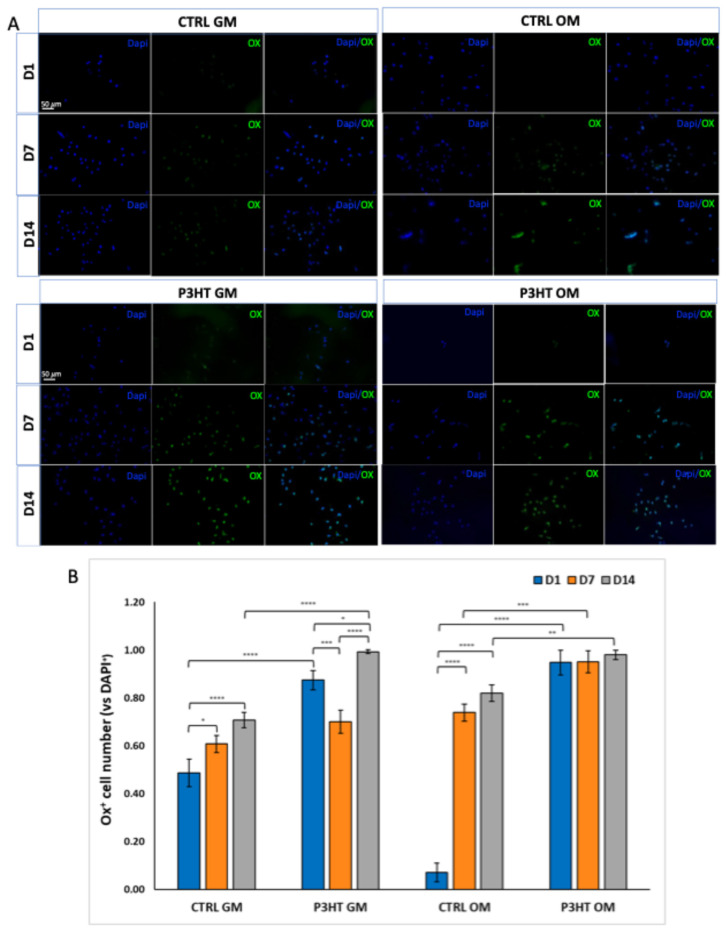
(**A**) Immunofluorescence analysis of hADMSCs cultured on P3HT thin polymer film, in presence of GM or OM for 1, 7 and 14 days, showing the expression of Osterix. Scale bars: 50 μm. (**B**) Quantitative evaluation of Osterix-positive cells vs. DAPI-positive cells per field. Multi-way ANOVA test *p*-value is reported and * *p* < 0.05, ** *p* < 0.01; *** *p* < 0.001, and **** *p* < 0.0001 show significant differences between the different groups, as reported by the Holm post hoc test.

Furthermore, according to qRT-PCR results, an immunofluorescence analysis performed with Osterix also displayed an increased expression over time, from 7 to 14 days, in hADMSCs grown on both P3HT GM and P3HT OM compared to the cells grown on CTRL slides in both culture media (Figure 5).

These results, in agreement with AR S data, suggest that P3HT polymeric thin films can promote osteoblastic differentiation of hADMSCs both in the presence and absence of osteoinductive culture medium, as demonstrated by the increasing expression levels of Osteopontin, Osterix, Osteocalcin, and Osteonectin. It has already been reported that Osterix and non-collagenous proteins, such as Osteopontin, Osteocalcin, and Osteonectin, are crucial in the regulation of cell proliferation and osteogenesis [53]. Osterix is an osteoblast-specific transcription factor that plays a crucial role in bone formation by activating several genes involved in the differentiation of preosteoblasts into mature osteoblasts and osteocytes [54]. Osteopontin is a major regulator of bone formation and mineralization, especially in bone turnover, and increases the proliferative capability of mesenchymal stem cells (MSCs) in a dose-dependent manner. [55]. Osteocalcin supports MSCs osteoblastic differentiation by increasing the extracellular matrix mineralization and ALP activity [56]. Osteonectin is crucial for the calcium release, and thereby influences matrix mineralization during bone formation [57].

### 3.4. Physico-Chemical Properties of the hADMSCs Cultured on P3HT Thin Polymer Film

**Figure 6:**
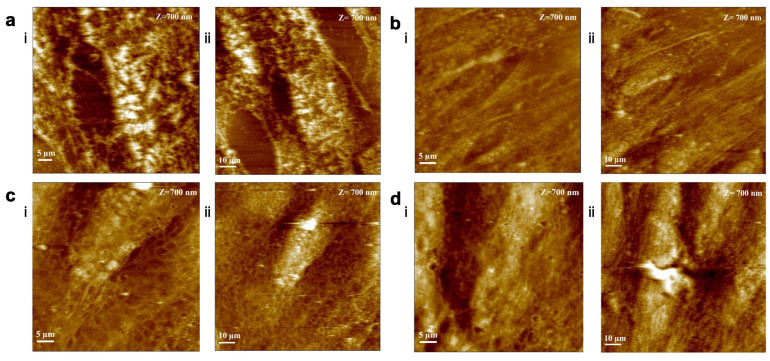
Morphological analysis of hADMSCs at D14 on glass with growth medium (GM) (**a**) and with osteogenic differentiation medium (OM) (**b**), on P3HT thin film in GM (**c**) and in OM (**d**). 50 µm × 50 µm acquisition (**i**) and 100 µm × 100 µm acquisition (**ii**).

hADMSCs cells were fixed after 14 days with PFA, and their morphology was investigated by atomic force microscopy (AFM) [58]. Figure 6 reports the height images of hADMSCs on glass with a normal growth medium (Figure 6(ai,aii)) and with an osteogenic differentiation medium (Figure 6(bi,bii)), and on P3HT with a normal growth medium (Figure 6(ci,cii)) and with an osteogenic differentiation medium (Figure 6(di,dii)), respectively.

Morphological analysis reveals a greater number of cells adhering to the polymeric surface when both media are present, in comparison to the control. Notably, on P3HT thin films exposed to the growth medium, the cells appear to be more uniformly spread, exhibit branching, and have smoother surfaces. in addition, the presence of stress fibers can be observed.

In order to investigate the effect of the substate on the extracellular matrix elasticity, mechanical properties were analyzed by force spectroscopy to investigate the effect on focal adhesion proteins expression, and of regulatory effects on mechanosignal transduction in osteoblasts [58,59,60,61,62].

Research indicates that the stress experienced by newly formed osteoblasts from various substrates, when subjected to the same mechanical external stimuli, arises from distinct adhesion mechanisms. This variation can result in differing levels of responsiveness among the osteoblasts.

**Figure 7:**
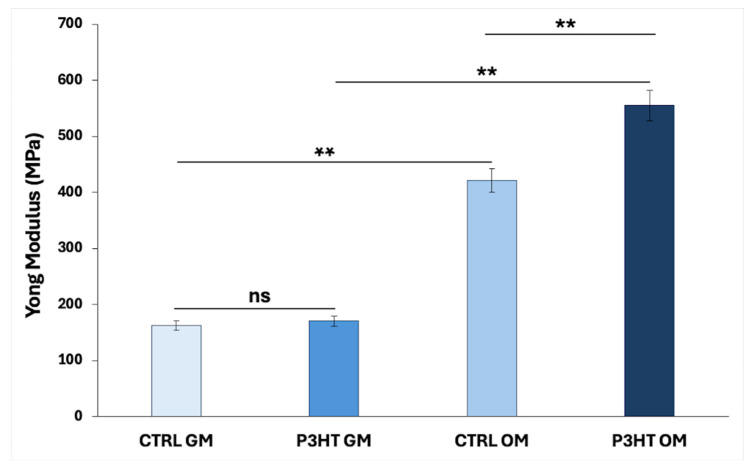
Young’s modulus of hADMSCs at D14 on glass (CTRL) and P3HT in presence of normal growth medium (GM) osteogenic differentiation medium (OM). The one-way ANOVA test *p*-value is reported and ** *p* < 0.01 show significant differences between the different groups, as reported by the Holm post hoc test. ns = not significant.

Figure 7 reports the cell Young’s modulus values. The substrate significantly influences the mechanical properties of the differentiated cells. In normal growth conditions, hADMSCs cells on P3HT show a slightly higher Young’s modulus value (~170.2 MPa) compared to the same conditions on glass (~162.2 MPa). Conversely, in osteogenic differentiation medium, the value on P3HT (~554.9 MPa) is higher than on glass (~421.6 MPa).

Biological findings indicate that P3HT thin films are capable of promoting matrix mineralization, especially when an osteogenic differentiation medium is present, leading to enhanced calcium deposition. The elevated Young’s modulus observed in the P3HT substrate can be attributed to the organization of the extracellular mineralized matrix and the accumulation of calcium deposits, which contribute to a more rigid cell membrane. In contrast, this notable difference is less pronounced in substrates maintained under standard growth conditions.

The findings indicate that the physicochemical properties of P3HT thin polymer films play a crucial role in promoting osteogenic differentiation by improving cell adhesion, proliferation, and extracellular matrix mineralization. This highlights their promise as bioactive scaffolds for bone tissue engineering.

## 4. Conclusions

Analyzing how living cells respond to conducting polymers is crucial for developing successful tissue engineering strategies. In this study, the cell behavior, cytoskeletal organization, and differentiation of hADMSCs on electroactive thin polymer films (P3HT) were investigated, both with a normal growth medium (GM) and with an osteogenic differentiation medium (OM).

The results showed that cells seeded on P3HT thin films presented a morphology similar to that of progenitor cells seeded on a reference glass substrate, suggesting that the polymer surface is biologically compatible with hADMSCs cells and promotes adhesion and proliferation both in the presence of GM and OM. It is worth noting that P3HT surfaces presented a very high value of mineralization in the presence of both GM and OM. In particular, the osteogenic differentiation medium induced calcium deposits, which led to the formation of a much stiffer extracellular matrix, which was confirmed by Young’s modulus measurements. Moreover, the increased expression of osteoblastic markers on cells grown on P3HT GM was more consistent than that detected on hADMSCs on P3HT OM. Our study suggests the possibility of using P3HT-covered substrates to produce cells displaying the osteoblast phenotype from human stem cells, without an external stimuli-mediated process, and even in the absence of osteogenic growth factor, by just considering the intrinsic properties of the material and the interactions established between the polymer and the cell biochemical signals. This system provides a versatile platform for next-generation biocompatible materials for regenerative medicine and tissue engineering, guiding the differentiation path with extreme operational ease.

In conclusion, the combined use of P3HT polymer and stem cells represents a promising strategy in bone regenerative medicine. A further understanding of the molecular mechanisms underlying osteogenic differentiation and the interaction between elastic polymers and stem cells is fundamental for the development of increasingly effective and targeted therapies for the treatment of pathological bone conditions.

## Figures and Tables

**Table 1:** Primer sequences.

Gene	Forward	Reverse
Osteopontin	AGTTTCGCAGACCTGACATCCAGT	TTCATAACTGTCCTTCCCACGGCT
Osteocalcin	GGCAGCGAGGTAGTGAAGAG	GATGTGGTCAGCCAACTCGT
Osteonectin	TAGCACACAGCCTACCACAAG	AGCAACTTCAGTCTGCTGAGGG
GAPDH	GCTCTCCAGAACATCATCCCTGCC	GCGTTGTCATACCAGGAAATGAGCTT

## Data Availability

The original contributions presented in the study are included in the article, further inquiries can be directed to the corresponding authors.
